# Effect of different surface treatments on the shear bond strength of nanofilled composite repairs

**DOI:** 10.15171/joddd.2016.002

**Published:** 2016-03-16

**Authors:** Ghazaleh Ahmadizenouz, Behnaz Esmaeili, Arnica Taghvaei, Zahra Jamali, Toloo Jafari, Farshid Amiri Daneshvar, Soraya Khafri

**Affiliations:** ^1^Assistant Professor, Department of Operative Dentistry, Faculty of Dentistry, Babol University of Medical Sciences, Babol, Iran; ^2^Dental Student, Faculty of Dentistry, Babol University of Medical Sciences, Babol, Iran; ^3^Assistant Professor, Department of Oral Medicine, Faculty of Dentistry, Tabriz University of Medical Sciences, Tabriz, Iran; ^4^Assistant Professor, Department of Prosthetic Dentistry, Faculty of Dentistry, Birjand University of Medical Sciences, Birjand, Iran; ^5^Assistant Professor, Department of Biostatistics and Epidemiology, Faculty of Medicine, Babol University of Medical Sciences, Babol, Iran

**Keywords:** Composite resin, dental air abrasion, dental restoration repair, Er:YAG lasers

## Abstract

***Background.*** Repairing aged composite resin is a challenging process. Many surface treatment options have been proposed to this end. This study evaluated the effect of different surface treatments on the shear bond strength (SBS) of nano-filled composite resin repairs.

***Methods.*** Seventy-five cylindrical specimens of a Filtek Z350XT composite resin were fabricated and stored in 37°C distilled water for 24 hours. After thermocycling, the specimens were divided into 5 groups according to the following surface treatments: no treatment (group 1); air abrasion with 50-μm aluminum oxide particles (group 2); irradiation with Er:YAG laser beams (group 3); roughening with coarse-grit diamond bur + 35% phosphoric acid (group 4); and etching with 9% hydrofluoric acid for 120 s (group 5). Another group of Filtek Z350XT composite resin samples (4×6 mm) was fabricated for the measurement of cohesive strength (group 6). A silane coupling agent and an adhesive system were applied after each surface treatment. The specimens were restored with the same composite resin and thermocycled again. A shearing force was applied to the interface in a universal testing machine. Data were analyzed using one-way ANOVA and post hoc Tukey tests (P < 0.05).

***Results.*** One-way ANOVA indicated significant differences between the groups (P < 0.05). SBS of controls was significantly lower than the other groups; differences between groups 2, 3, 4, 5 and 6 were not significant. Surface treatment with diamond bur + 35% phosphoric acid resulted in the highest bond strength.

***Conclusion.*** All the surface treatments used in this study improved the shear bond strength of nanofilled composite resin used.

## Introduction

 Composite resins have significantly improved over the last decades; however, failures may occur as a result of discoloration, secondary caries, margin ditching or simply fractures.^1-4^ Treatment choices are repairing or replacing the whole restoration.^1,5-9^ Replacing a deficient restoration completely results in overextension of the preparation,^10^ loss of sound tooth structure and increased risk of pulpal exposure.^3,7-9,11^ According to several clinical studies, repairing the pre-existing restoration is a more conservative alternative that can increase the longevity of the restoration, preserve the sound tooth structure and reduce operative trauma.^4,5,12^

 In clinical practice, bonding between two composite layers is accomplished by the presence of an oxygen-enriched surface layer that remains unpolymerized.^3,5,7,13^ This layer contains unreacted C=C bonds, allowing the monomers of the new composite resin to bond to it.^7,13,14^In an aged composite resin the adhesion to a new one reduces 25% to 80% of its original cohesive strength due to a diminished amount of unreacted double bonds.^1,12,15^ The success of new composite-to- old composite resin adhesion depends on the chemical composition of the surface, roughness, wetting and the surface conditioning methods applied.^7,12,13^ Therefore, different surface treatment modalities have been used to enhance the repair bond strength of composite resins,^1,2,4,7-9,16^including bur roughening, etching with hydrofluoric or phosphoric acids, air abrasion, silica coating and silanization.^4,6-9,15,16^ In recent years there has been more focus on the efficiency of lasers for composite repair bond strength, including Er:YAG laser.^4,8,9,17^

 Studies have shown that Er:YAG laser can influence the surface of composite resins in addition to tooth surfaces.^4,8,9,17^ The wavelength of Er:YAG laser is 2940 nm and it is absorbed by the water and hydroxyapatite of the tooth. In the laser ablation process the produced heat releases hydroxyl groups from hydroxyapatite,^18^ causing the water surrounding the apatite crystals to evaporate suddenly. This evaporation results in an increase in the internal tissue pressure and subsequently micro-explosions happen.^19,20^ Most of the energy is used during the ablation process and the rest diffuses into the adjacent tissues, without an extreme increase in temperature.^21,22^ Additionally, the use of laser on enamel and dentin results in micro-retentions on the enamel and opening of the dentinal tubules.^19^

 In an attempt to achieve long-lasting composite restorations, the composition of composite resins has been modified in recent years. Major modifications include changes in the size and distribution of fillers with reduced filler particle sizes and increased loading. This has led to the development of nanofilled composite resins.^7,13^ The range of the filler size in nanofilled composite is between 5 and 100 nm and the particles are in clusters or dispersed forms.^13^ The repair of nanofilled composite resins has not been yet investigated in detail and there is no consensus on the results obtained with the different surface treatments.^1,11-13^

 Therefore, the aim of this study was to evaluate the effect of different mechanical and chemical surface treatment procedures on shear bond strength of repaired nanofilled composite resin and to characterize changes in surface topography following each treatment. The null hypothesis tested was that different surface treatments would not affect the shear bond strength.

## Methods

 No ethical approval was obtained because this in vitro study only involved non-invasive procedures on composite resin samples. The brands, manufacturers and chemical compositions of the materials used in this study are listed in Table 1.

**Table 1:** List of brands, manufacturers and chemical compositions of the materials used.

**Material**	**Manufacture**	**Chemical composition**
**Filtek Z350 XT Universal Restorative** **(shades A2 and A4)**	3M ESPE, St. Paul, MN, USA	Bis-GMA, UDMA, TEGDMA, PEGDMA, Bis-EMA, non-aggregated 4 to 10nm zirconia, non-aggregated 20 nm silica and aggregated zirconia/silica cluster filler (63.3 vol%)
**Swiss TEC SL Etchant Gel**	Coltene Whaledent AG, Altstätten Switzerland	35% phosphoric acid
**Porcelain Etch Gel**	PULPDENT Corp*,* Watertown, MA , USA	9% Hydrofluoric Acid
**Adper Single Bond2**	3M ESPE, St. Paul, MN, USA	Dimethacrylate, HEMA, polyalcenoic acid copolymer, silane treated colloidal silica, ethanol, water, photoinitiator
**Silane Bond Enhancer**	PULPDENT Corp, Watertown, MA , USA	3-methacryloxypropyltrimethoxysilane
**Diamond Bur**	FGS110012, DIA-ITALY, ITALY	Grit:100µm

###  Sample preparation

 Seventy-five cylindrical specimens, 4 mm in height and 4 mm in diameter, were prepared by the layering technique with 2-mm-thick increments of a nanofilled composite resin (Filtek Z350 XT, shade A2) using plastic molds. Each layer was light-cured for 20 s with an LED light-curing unit (Valo, Ultradent Products, Inc. UT, USA) according to the manufacturer’s instructions. The intensity of the light-curing unit was 1000 mW/cm^2^ and verified by a radiometer after every 5 specimens. The last increment was covered with a Mylar strip (KerrHawe SA, Bioggio, Switzerland) and a glass slide in order to create a smooth surface and to prevent the formation of an oxygen-inhibited layer. After polymerization, the molds were gently removed and the specimens were cured from each side for 20 s in order to ensure uniform and complete polymerization. Fifteen additional specimens, 6 mm in height and 4 mm in diameter, were prepared in the same manner in order to evaluate the cohesive strength. To age the composite resin, the substrates were placed in distilled water at 37°C for 24 hrs and then thermocycled for 500 cycles at 5 ± 2/55 ± 2°C with a dwell time of 30 s and transfer time of 10 s.^23^

 Except for the samples of the cohesive group (group 6), the other specimens were randomly divided into five groups (N=15) according to the surface treatment applied.

 In group 1 (control), no surface treatment was performed on the specimens.

 In group 2, the samples were air-abraded at a pressure of 60 PSI using an air abrasion device (AEROETCHER, D670, PARKELL Farmingdale, NY, USA) for 5 s with 50-µm aluminum oxide particles. The tip was positioned 5 mm away from the target and perpendicular to the specimen surface. Subsequently, the specimens were rinsed under tap water and air-dried.

 In group 3, composite resin surfaces were irradiated with Er:YAG laser beams (Doctor Smile, LAEDL001.1, LAMBDA Scientifica S.p.A, Italy). A H6/12-type laser tip was used for surface treatment. Laser energy was delivered in pulse mode at a wavelength of 2.94 µm, a duration of 75 µs and a repetition rate of 25 Hz. The output power was 1.5 W at 60% air level and 30% water level. The beam was perpendicular to the target area, with a distance of 1 mm between the laser tip and the composite resin surface. Subsequently, the specimens were rinsed and air-dried.

 In group 4, composite resin surfaces were roughened in three strokes with a coarse diamond bur using a high-speed handpiece with water spray. A new diamond bur was used for each 5 samples. Then 35% phosphoric acid was applied for 15 s and washed with water and dried.

 Finally, in group 5 the substrates were etched with 9% hydrofluoric acid (HF) (Porcelain Etch Gel) for 120 s, rinsed and dried.

 After surface treatments, silane coupling agent (Silane Bond Enhancer) was applied to all the specimens in a thin layer, the solvent was gently removed under compressed air. Thereafter, Single Bond 2 bonding agent was applied on sample surfaces according to the manufacturer’s instructions and light-polymerized for 20 s using a Valo LED light-curing unit at a light intensity of 1000 mW/cm^2^.

 Then cylindrical molds (2×2 mm) were placed at the center of the specimens and filled with the A_4_ shade of Filtek Z350XT composite resin by the same operator and light-cured for 20 s. The molds were then removed and additional curing was carried out for 20 s from each side.

 All the specimens were stored in 37°C distilled water for 24 hrs and additionally thermocycled for 500 cycles at 5±2/55±2°C with a dwell time of 30 s and a transfer time of 10 s.

 The specimens were mounted in acrylic resin and placed in a universal testing machine (Zwick ROELL Z050, Germany) and a shear force was applied using a shearing blade parallel to the adhesive interface. The load was applied to the interface at a cross-head speed of 1 mm/min until failure and the stress-strain curve was analyzed with the machine’s software program. The same technique was used to measure the cohesive strength of the samples in group 6.

 In order to visualize the topography of samples after the surface treatment, one specimen from each group was selected and gold-sputtered by a 150-A^°^ thin gold layer; the surface topography was then‏ evaluated under a scanning electron microscope (Tescan Vega-II; Tescan, S.RO. LibusiniaTrida, CZ) at ×1000 magnification and kVp=15.

 The failure modes of the specimens were determined at ×40 under a stereomicroscope (Motic Smz-143 SERIES, Micro-optic industrial group Co, Xiamen, China) and recorded as ‘cohesive in aged or new composite’, ‘adhesive at the interface’, or ‘mixed adhesive-cohesive’.

###  Statistical analysis

 Data were collected and analyzed with SPSS V.20. Analyses were performed by one-way ANOVA and post hoc Tukey tests. Statistical significance was defined at α = 0.05.

## Results

**Figure 1:**
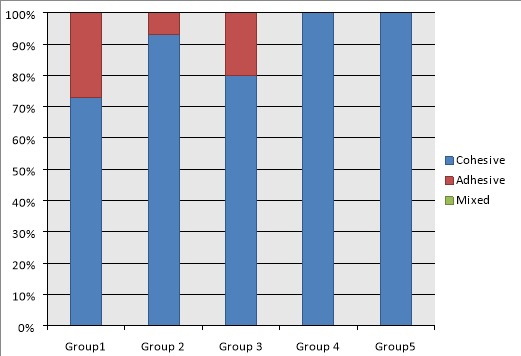
Percentages of fracture modes evaluated under a stereomicroscope after shear bond strength testing of each group (Group 1: control, group 2: air abrasion, group 3: Er:YAG Laser, group 4‏: diamond bur + phosphoric acid, group 5: HF acid).

 The means and standard deviations of repair shear bond strengths in the study groups are presented in Table 2. The highest shear bond strength was found in group 4‏ (diamond bur + phosphoric acid) and the lowest in group‏ 1 (control). One-way ANOVA indicated significant differences between the study groups (P < 0.001). Two-by-two comparisons of the groups revealed significant differences in repair bond strength between group 1 and the other five study groups; however, there were no statistically significant differences between groups 2, 3, 4, 5 and 6. The percentages of fracture modes of the samples are illustrated in Figure 1. The mode of failure was predominantly cohesive for all the groups. Only a few fractures were adhesive and there were no mixed failures.

**Table 2:** Means and standard deviations of shear bond strengths in the studied groups (in MPa)

**Groups**	**N**	**Mean (MPa)**	**Std. Deviation**	**Min (MPa)**	**Max (MPa)**
**1**	15	20.22 ^a^	5.12	11.96	31.68
**2**	15	32.29 ^b^	5.42	25.39	44.09
**3**	15	29.14 ^b^	3.43	22.13	35.47
**4**	15	35.51 ^b^	4.41	25.49	44.56
**5**	15	33.77 ^b^	4.67	26.63	42.22
**6**	15	27.79 ^b^	5.70	18.37	37.88

Group 1: control; group 2: air abrasion; group 3: Er:YAG laser; group 4::diamond bur + phosphoric acid; group 5: HF acid; group 6: bulk. Different letters in a column indicate the statistically significant differences at α=0.001 between the two groups.

###  SEM analysis

**Figure 2:**
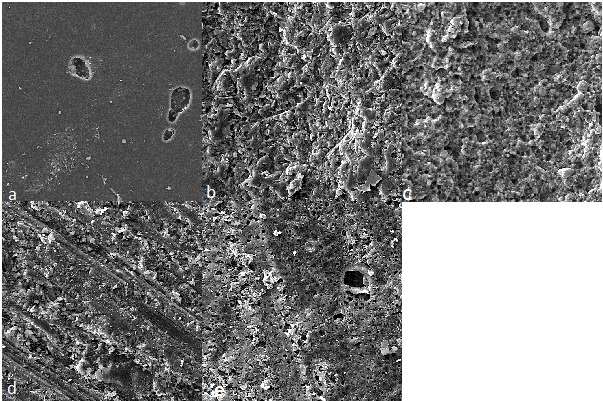
Scanning electron micrographs of the aged resin composite. (a) control sample with no surface treatment; (b) air abraded sample; (c) Er:YAG laser-treated sample; (d) bur + acid etched sample; (e) HF-treated sample.

Figure 2 illustrates SEM micrographs of Filtek Z350XT composite resin surfaces treated with various techniques. As determined from the SEM micrographs, the control sample had a relatively smooth surface. The bur-treated and acid-etched sample exhibited a rougher surface and more area for micromechanical retention compared to other treatments. In the laser-treated samples, a homogeneous micro-retentive feature was noticeable. In HF-etched surfaces, the specimen exhibited a moderate amount of surface relief along with pores. In sand-blasted samples, a rough pattern was visible along with grooves.

## Discussion

 Adhesion of a new composite resin to an aged one is challenging because of the absence of oxygen-inhibited layer and reduction of unsaturated C=C bonds.^3,11,15^ A variety of surface treatments have been used to improve the repair bond strength of composite resins.^1,2,4,7^ In the present study, four different surface treatment methods were evaluated to achieve optimal repair bond strength. According to the results, the lowest repair bond strength was recorded in the control group, which was expected due to smooth surface visible in scanning electron micrograph of the sample in this group. This is in consistent with the results of other studies,^6,13,17^and indicates the importance of surface roughening in improving the repair bond strength of nanofilled composite resins. All the surface treatments applied in this study reached the cohesive strength of the en bloc samples and the repair bond strengths were significantly higher than those of the controls. Inclusion of both the control and cohesive groups in the present study defines the influence of different methods on repair bond strength and the repair potential compared to the cohesive strength of the material.^10^

 Based on our results, roughening the aged composite resin with a coarse bur and subsequent acid etching formed the highest repair bond strength, followed by hydrofluoric acid etching, air abrasion and Er:YAG laser, although no significant differences were observed between the groups. This indicates that all the four surface treatment modalities were effective in bonding the aged composite resin to the fresh one. Thus, the null hypothesis was rejected.

 In the present study, after surface treatments and prior to the bonding with all the specimens, a silane solution was applied. Composite materials roughly have 50 vol% filler; therefore, 50% of the roughened composite surface should consist of fillers.^3^ Silane molecules have two main functional groups; the silanol bonds to the silica particles of a composite resin and at the same time the organofunctional group of this compound bonds to the methacrylate of the bonding agent.^3,10,15^ Silane also assists the infiltration of bonding agent by increasing the wettability of the surface.^5,7,10,13-16,23,24^

 Application of a diamond bur and subsequent phosphoric acid etching in the present study yielded the highest repair bond strength. This finding is supported by a study of Tabatabaei et al.^23^ This method of surface treatment creates macro-retentive as well as micro-retentive features and this may differentially expose filler particles.^23^ This finding is supported by the SEM image which shows a more retentive linear patterns and courser surface. Moreover, acid etching removes the smear debris and exposes the underlying surface and fillers. This results in an increased surface area which can help stress distribution along the interface of the two bonded substrates.^11^Additionally acid etching might also set off the reaction between a silane coupling agent and silica surface.^10^ A combination of bur roughening, phosphoric acid etching and silane application can be suggested to achieve higher repair bond strength in Filtek Z350XT composite resin.

 According to our results, air abrasion with 50-μm aluminum oxide particles produced favorable repair bond strength in the aged composite resin. Following air abrasion, some of the resin matrix is removed and the surface fillers are exposed resulting in an increased surface roughness of the composite resin.^8,23^Several previous studies have reported contradictory findings about air abrasion. In some studies, sandblasting promoted the best repair bond strength,^1,25,26^ while a reduction in repair strength after surface abrasion was found in a few studies.^8,27,28^ This reduction has been ascribed to the exposure of filler particles, and hence decreased amount of available resin for bonding.^8,23^ It seems that the application of a silane coupling agent following sandblasting in the present study enhanced the bond to the exposed filler particles and thus, increased the repair bond strength. In the present study, evaluation of scanning electron microscope images of the air-abraded samples revealed an increase in surface roughness in a pattern different from other treatment modalities. It has been reported that the surface characteristics following air abrasion depends on the microstructure and composition of the material. In nanofilled composite resins, breaking off of the clusters occurs when they are subjected to abrasion.^14^ Thus the loss of fillers might reduce the interaction with silane compared to diamond bur-treated groups. Moreover after air abrasion the smear debris is not removed and this may reduce the surface area available for bonding, hence reducing the bond strength compared to the acid etching group.

 In the present study, hydrofluoric acid treatment resulted in significantly higher SBSs than the control group, which is consistent with the findings of other studies.^10,29^ HF acid dissolves the glass particles of the composite, leaving micro-mechanical pores that allow adequate bonding agent infiltration.^29^ SEM analysis of samples etched with HF revealed a moderate amount of surface relief with partial removal of filler particles and the presence of pores. The fillers of Filtek Z350 XT are a combination of silica fillers, zirconia fillers and zirconia/silica cluster fillers. It seems that subsequent to etching with HF, silica-containing fillers are partially dissolved and the remaining fillers react with silane agent, promoting the bond, in addition to micro-mechanical retention. However, other composite repair studies have reported that the HF etching of the composite surface decreased the repair bond strength.^1,30,31^ This difference can be due to differences in the type of composite resins used in these studies. The effect of HF is related to the percentage, size and type of the inorganic filler of composite resin.^1^ HF etching can be an effective surface treatment but necessitates extreme care when used for intraoral repairs due to the risk for acid burns and soft tissue necrosis.^10^

 The results of this study showed that SBS of samples in the laser group was significantly higher than that of the controls, but in comparison to diamond bur, air abrasion and HF groups lower bond strengths were achieved, although the difference was not significant. This finding is consistent with that of Rosatto et al,^9^ in which the Er:YAG laser yielded results similar to diamond bur and sandblasting. Likewise, Bektas et al^4^ concluded that repair bond strength of laser-treated surfaces was comparable to that of bur-treated surfaces. However, in the study of Alizadeh et al^32^ with Er,Cr:YSGG laser, and Hasan^8^ with Er:YAG laser, higher repair bond strength was reported for the laser groups compared to other surface treatments. The differences might be related to the type, structure and chemical composition of composites used as well as laser parameters that affected the efficacy of mechanical surface treatments.^4^

 Electron microscope images in the present study revealed that laser irradiation resulted in formation of a micro-retentive pitting feature, without smear layer formation, which increases the bonding surface, resulting in a higher repair bond strength compared to the controls. However, the micro-retentive feature was less prominent compared to other treatment modalities. Lizarelli et al^33^ reported that the micromorphology of the laser-irradiated surface depends on the chemical composition and structure of composite resin. Composite resins with greater filler-matrix bond energy and cohesion are more resistant to laser ablation. Under laser ablation the polymeric matrix is abraded first and subsequently the filler particles are released. It seems that in Filtek Z350XT nanofilled composite resin, presence of nanoparticles and nanoclusters increases the filler loadings and leads to less matrix exposed for ablation.

 Bond strength between 15 MPa to 25 MPa is suggested for composite resin repairs in some studies. These values are typical of the bond strength of composite resin to dentin,^23,34^ which could be clinically accepted. In our study, all the repair groups reached these values, with even the control group. It seems that high repair bond strength in Filtek Z350XT nanofilled composite resin is achievable by any of the treatment modalities. This could be explained by the fact that Filtek Z350XT composite resin consists of nano-sized silica particles (20 nm) and clusters of Si/Zr. Small filler particles expose a higher surface area and increase the bonding substrate. It is also believed that the nanoclusters may present a reinforcing mechanism and that the silane infiltration within the intimacy of the nanoclusters modifies the response to loading stresses, thus providing an improved clinical performance.^35^

 A general repair technique cannot be suggested for nanofilled composite resins since all the surface treatments showed higher SBS than the cohesive controls and can be considered appropriate. However, there is a limitation in utilizing these methods clinically; for example HF is corrosive for intraoral use, aerosols in air abrasion can be harmful for respiratory system and Er:YAG laser needs special equipment and proficiency. Bur roughening and acid etching on the other hand can be a safe and cost-effective alternative and should be recommended to be used clinically for repairing nanofilled composite resins. Our study was carried out in vitro; therefore, it is difficult to extend the results to clinical situations. It is suggested that in future studies the repair bond strength of nanofilled composites be evaluated in vivo where they are exposed to the effects of pH and temperature changes, salivary enzymes and the oral environment.

## Conclusion

 Within the limitations of this study, it was concluded that composite resin surface treatment with bur and acid etching, air abrasion, HF acid and Er:YAG laser resulted in similar bond strength and can be recommended to obtain optimal repair bond strength.

## Acknowledgments

 The authors thank Babol University of Medical Sciences for supporting this research. The authors are grateful to Dr. Evangeline Foronda for the English proofreading.

## Authors’ contributions

 The study was planned by GA, BE and AT. AT carried out the laboratory procedures and shear bond testing process. The statistical analyses and interpretation of data were carried out by SK. GA, BE, AT and TJ were responsible for manuscript preparation. ZJ, TJ, and FAD critically revised the manuscript for intellectual content. All authors contributed to the final draft, and have read and approved the final manuscript.

## Funding

 This study was a part of a thesis and research project (Grant No: 1710) supported and funded by Babol University of Medical Sciences.

## Competing interests

 The authors declare that they have no competing interests with regards to the authorship and/or publication of this article.

## Ethics approval

 Not applicable.
